# Utility of Vascular Flow Pattern Evaluation Using Color Doppler, Power Doppler, and Microvascular Imaging in the Diagnosis of Cervical Lymph Node Diseases

**DOI:** 10.7759/cureus.110878

**Published:** 2026-06-15

**Authors:** Prashant Onkar, Sanhita V Patil, Dipali Kadam, Suresh Phatak, Kajal Mitra, Jyotiprakash Sharma, Ashish N Ambhore, Sandip Dhote, Jitendra B Sahu, Mayank Rangari

**Affiliations:** 1 Radiodiagnosis, NKP Salve Institute of Medical Sciences and Research Centre, Lata Mangeshkar Hospital, Nagpur, IND

**Keywords:** cervical lymphadenopathy, color-doppler ultrasound, metastatic malignant lymphadenopathy, microvascular imaging, power doppler ultrasound

## Abstract

Background: Vascular flow patterns within cervical lymph nodes vary significantly across inflammatory and neoplastic conditions.

Objective: The objective of the study is to evaluate the role of color Doppler, power Doppler, and microvascular imaging (MVI) in characterizing lymph node vascularity and differentiating inflammatory from neoplastic lymphadenopathy.

Methods: Thirty-five patients with palpable cervical lymphadenopathy underwent neck ultrasonography with gray-scale imaging, color Doppler, power Doppler, and MVI assessment. Fine-needle aspiration cytology (FNAC) was subsequently performed for pathological confirmation.

Results: MVI demonstrated superior sensitivity (75.0% vs. 41.67%), negative predictive value (88.46% vs. 76.67%), and overall diagnostic accuracy (91.43% vs. 80.0%) compared with color and power Doppler techniques in detecting microvascular flow in cervical lymph nodes. Inflammatory lymph nodes predominantly exhibited central hilar vascularity or avascular patterns, whereas malignant nodes showed peripheral or mixed vascularity with evidence of neovascularization.

Conclusion: MVI significantly enhances diagnostic accuracy in evaluating cervical lymph nodes and provides superior microvascular visualization over conventional Doppler techniques and, hence, aids in identifying the most suspicious nodes for targeted FNAC or biopsy.

## Introduction

Cervical lymphadenopathy arises from a wide spectrum of inflammatory, infectious, benign, and malignant conditions, and alterations in lymph node vascular architecture often reflect the underlying pathology [1]. Reactive or inflammatory lymph nodes usually preserve normal architecture with predominant central or hilar vascularity, whereas malignant lymph nodes tend to demonstrate peripheral or irregular neovascularization due to tumor infiltration and angiogenesis [2,3]. High-frequency ultrasound allows detailed assessment of nodal morphology, while color Doppler and power Doppler imaging (PDI) provide valuable information regarding intranodal vascular distribution and flow patterns [4]. However, conventional Doppler techniques have limited sensitivity for slow-flow and microvascular signals, which may reduce accuracy in detecting malignant neovascularity. Microvascular imaging (MVI) has shown improved visualization of peripheral and irregular microvascular flow patterns in metastatic cervical lymph nodes compared with conventional Doppler techniques [5]. It addresses these limitations by enabling visualization of low-velocity microvasculature and detailed intranodal vascular architecture without contrast administration [6]. Recent studies indicate that MVI improves diagnostic confidence and demonstrates superior performance compared with conventional Doppler techniques in differentiating benign from malignant cervical lymphadenopathy [7]. The present study compares these ultrasound modalities using fine-needle aspiration cytology (FNAC) as the reference standard.

Preliminary results of this original research work were presented as a paper presentation at Sono Summit 2025 held in June 2025 in Chennai, India. The presentation focused on the diagnostic value of gray-scale ultrasonography, Doppler evaluation, and MVI in the characterization of lymph node disease.

## Materials and methods

Study design

A prospective observational study was conducted over a period of six months, from February 1, 2025, to July 31, 2025, in the Department of Radiodiagnosis at Lata Mangeshkar Hospital, Nagpur, India.

Study population

An a priori sample size calculation was performed, and a total sample size of 35 cases was determined and included in the study. Patients across all age groups and of both sexes presenting with clinically palpable cervical lymphadenopathy who were referred for ultrasonographic evaluation of cervical lymph nodes and who provided written informed consent were included in the study. Patients with a history of prior neck surgery or lymph node excision and those who had received chemotherapy or radiotherapy for head and neck malignancy were excluded from the study. Patients who did not provide informed consent were also excluded.

Ultrasound equipment

All ultrasonographic examinations were performed using a Samsung V7 (Samsung Medison Co., Ltd, Seoul, South Korea) ultrasound system equipped with a high-frequency linear transducer operating within a frequency range of 5-14 MHz. High-resolution ultrasound was utilized for the evaluation of cervical lymph nodes. Gray-scale ultrasonography, color Doppler, power Doppler, and MVI in both color and power modes were employed to assess nodal morphology and vascularity.

Imaging protocol

Each cervical lymph node was systematically evaluated using gray-scale ultrasonography for morphological characteristics, including size, shape, border definition, and the presence or absence of an echogenic hilum. Vascular assessment was subsequently performed using color Doppler, power Doppler, and MVI (MVI-both color and power modes) techniques.

Image acquisition and interpretation were performed by two radiologists (a radiologist and a senior experienced radiologist), and a final diagnosis was reached by consensus discussion. However, no formal statistical assessment of interobserver agreement was performed. Based on the observed vascular distribution, lymph nodes were categorized into central, peripheral, avascular, neovascularization, or mixed vascularity patterns.

Reference standard

FNAC was used as the reference standard for the confirmation of the nature of cervical lymph nodes. Cytopathological examination of FNAC samples was performed to differentiate benign/reactive lymphadenitis from malignant lymph node involvement, and the ultrasonographic imaging findings were prospectively correlated with the FNAC diagnosis.

Statistical analysis

Sensitivity, specificity, positive predictive value (PPV), negative predictive value (NPV), and overall diagnostic accuracy (along with their respective 95% confidence intervals) were calculated for color and power Doppler and MVI modalities. The ultrasonographic findings were correlated with FNAC results to assess the diagnostic performance of the imaging technique in differentiating inflammatory and neoplastic cervical lymph nodes.

Ethical considerations

The study was approved by the Institutional Ethics Committee prior to commencement. Written informed consent was obtained from all participants before enrollment. The study was conducted in accordance with the ethical principles of the Declaration of Helsinki, and patient confidentiality was maintained throughout the study.

## Results

A total of 35 cervical lymph nodes were assessed, of which 23 were of inflammatory and 12 were of neoplastic etiology.

Vascular patterns observed

Inflammatory or reactive lymph nodes predominantly demonstrated a central vascular pattern with dilated hilar vessels and increased overall vascular flow. Tuberculous lymph nodes most commonly exhibited an avascular pattern, although a few nodes showed peripheral vascularity. Neoplastic lymph nodes predominantly demonstrated peripheral vascularity and irregular neovascularization, with some cases exhibiting a mixed vascular pattern.

Of the 35 lymph nodes evaluated, color and power Doppler classified 30 (85.7%) as inflammatory and five (14.3%) as neoplastic. MVI categorized 26 (74.3%) lymph nodes as inflammatory and nine (25.7%) as neoplastic. Based on FNAC, 23 (65.7%) lymph nodes were diagnosed as inflammatory and 12 (34.3%) as neoplastic. A greater number of neoplastic lymph nodes were identified by MVI compared to color and power Doppler, with the FNAC findings serving as the reference standard (Table 1).

**Table 1:** Comparison of diagnostic categorization by color and power Doppler ultrasound, microvascular imaging, and fine-needle aspiration cytology (FNAC) in cervical lymphadenopathy.

Total = 35	Color and power Doppler ultrasound	Microvascular imaging	FNAC
Inflammatory	30	26	23
Neoplastic	5	9	12

MVI demonstrated higher sensitivity than color and power Doppler ultrasound (75.0% vs. 41.67%), correctly identifying a greater number of neoplastic lymph nodes (9 vs. 5). Both modalities exhibited specificity and PPV of 100%, with no false-positive diagnoses. Additionally, MVI showed a higher NPV (88.46% vs. 76.67%) and overall diagnostic accuracy (91.43% vs. 80.0%) compared with color and power Doppler ultrasound. MVI also resulted in fewer false-negative cases, indicating improved detection of neoplastic lymph node involvement (with 95% confidence intervals as provided) (Table 2).

**Table 2:** Diagnostic performance of color and power Doppler ultrasound and microvascular imaging (MVI) for the diagnosis of neoplastic cervical lymph nodes. CI: confidence interval

Parameter	Color and power Doppler ultrasound	95% CI (color and power Doppler)	MVI	95% CI (MVI)
True positive (TP)	5	-	9	-
True negative (TN)	23	-	23	-
False positive (FP)	0	-	0	-
False negative (FN)	7	-	3	-
Sensitivity	41.67%	19.3%-68.1%	75.00%	46.8%-91.1%
Specificity	100%	85.7%-100%	100%	85.7%-100%
Positive predictive value (PPV)	100%	56.6%-100%	100%	70.1%-100%
Negative predictive value (NPV)	76.67%	59.1%-88.2%	88.46%	71.0%-96.0%
Diagnostic accuracy	80.00%	64.1%-90.0%	91.43%	77.6%-97.0%

Both modalities demonstrated sensitivity and NPV of 100%, indicating that all inflammatory lymph nodes confirmed on FNAC were correctly identified. However, MVI showed higher specificity (75.0% vs. 41.67%), PPV (88.46% vs. 76.67%), and overall diagnostic accuracy (91.43% vs. 80.0%) compared with color and power Doppler ultrasound. Furthermore, MVI yielded fewer false-positive diagnoses (3 vs. 7), indicating better discrimination between inflammatory and neoplastic lymph nodes (with 95% confidence intervals as provided) (Table 3).

**Table 3:** Diagnostic performance of color and power Doppler ultrasound and microvascular imaging (MVI) for the diagnosis of inflammatory cervical lymph nodes. CI: confidence interval

Parameter	Color and power Doppler ultrasound	95% CI (color and power Doppler)	MVI	95% CI (MVI)
True positive (TP)	23	-	23	-
True negative (TN)	5	-	9	-
False positive (FP)	7	-	3	-
False negative (FN)	0	-	0	-
Sensitivity (TP/(TP + FN) × 100)	100.0%	85.7%-100.0%	100.0%	85.7%-100.0%
Specificity (TN/(TN + FP) × 100)	41.67%	19.3%-68.1%	75.00%	46.8%-91.1%
Positive predictive value (PPV) (TP/(TP + FP) × 100)	76.67%	59.1%-88.2%	88.46%	71.0%-96.0%
Negative predictive value (NPV) (TN/(TN + FN) × 100)	100.0%	56.6%-100.0%	100.0%	70.1%-100.0%
Diagnostic accuracy ((TP + TN)/total × 100)	80.00%	64.1%-90.0%	91.43%	77.6%-97.0%

Diagnostic performance summary

MVI detected microvascular flow with greater clarity than color and power Doppler, particularly in low-flow or fine vascular structures. MVI improved visualization of flow patterns that correlated strongly with FNAC results, hence showing the highest sensitivity, specificity, and overall accuracy as compared to color and power Doppler. Representative cases demonstrating characteristic findings on gray-scale ultrasound, color Doppler, power Doppler, MVI, and corresponding histopathological examination (HPE) are presented below.

**Figure 1:**
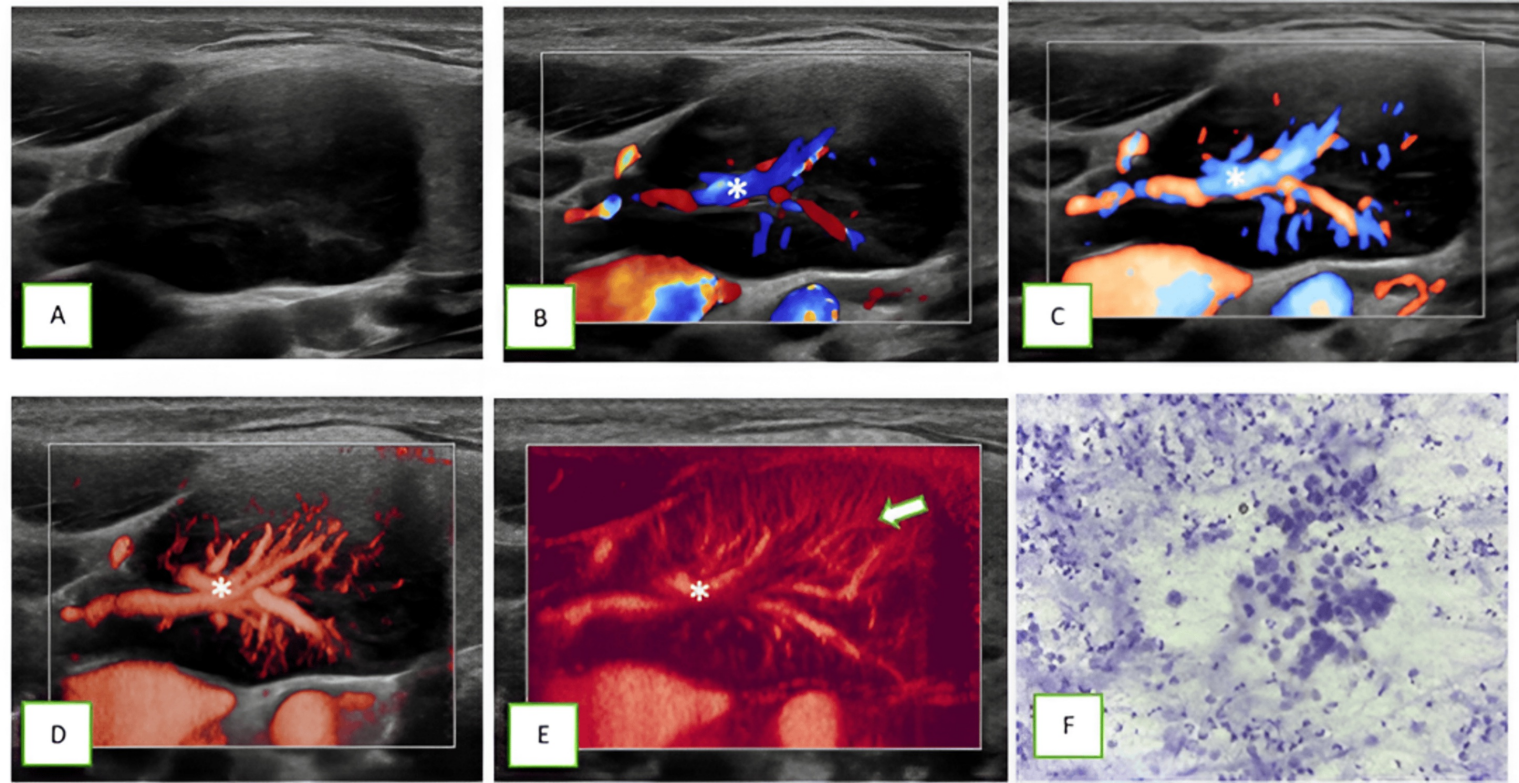
Reactive lymphadenitis. A 35-year-old woman presented with fever, mild throat pain, and bilateral palpable cervical lymph nodes. (A) Gray-scale ultrasound showed a poorly visualized hilum. (B-E) Color, power Doppler, MVI (color), MVI (power), respectively, demonstrated prominent hilar vessels (*) with enhanced detection of small vessels by MVI, highest by MVI (power mode) (white arrow)-suggesting an inflammatory etiology. (F) Corresponding cytology showed reactive lymphoid cells consistent with reactive lymphadenitis. MVI: microvascular imaging

Figure 1 shows a case of reactive lymphadenitis demonstrating a prominent central hilar vessel (*) with increased overall vascularity, wherein small intranodal vessels were more conspicuously revealed on MVI, particularly on MVI-power mode (white arrow in Figure 1E), highlighting its superior sensitivity for detecting low-flow microvascular signals.

**Figure 2:**
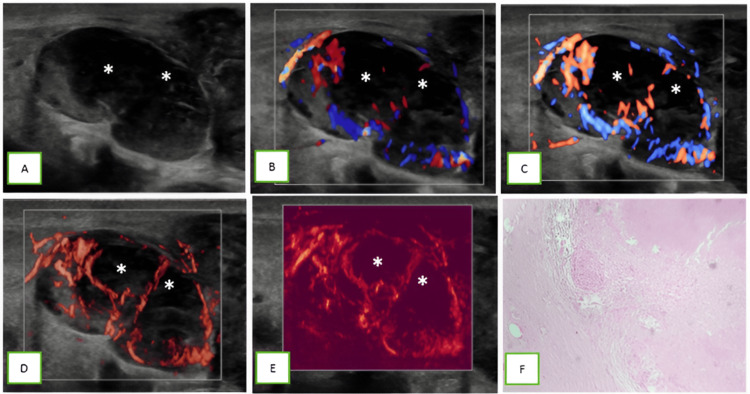
Tubercular lymphadenitis. A 23-year-old patient presented with evening rise of temperature, weight loss, and palpable bilateral cervical lymph nodes. (A) Gray-scale ultrasound (US) shows an enlarged hypoechoic cervical lymph node with central necrosis and loss of normal hilar architecture. (B-E) Color Doppler, power Doppler, MVI (color), and MVI (power), respectively, revealed necrotic areas (*) with predominantly peripheral vascularity, while MVI demonstrates enhanced peripheral vessels outlining the necrotic areas and distorted vascular architecture-suggesting tubercular etiology. (F) Histopathological examination demonstrated well-formed epithelioid granulomas with surrounding chronic inflammatory infiltrate and areas of central caseous necrosis, consistent with tuberculous lymphadenitis. MVI: microvascular imaging

A case of tuberculous lymphadenitis is demonstrated in Figure 2, which predominantly shows central avascular areas (*), likely representing necrosis, while MVI (significantly in the MVI power mode-Figure 2E) demonstrated enhanced delineation of peripheral microvessels outlining the necrotic regions along with distorted vascular architecture, thereby improving visualization of characteristic vascular changes.

**Figure 3:**
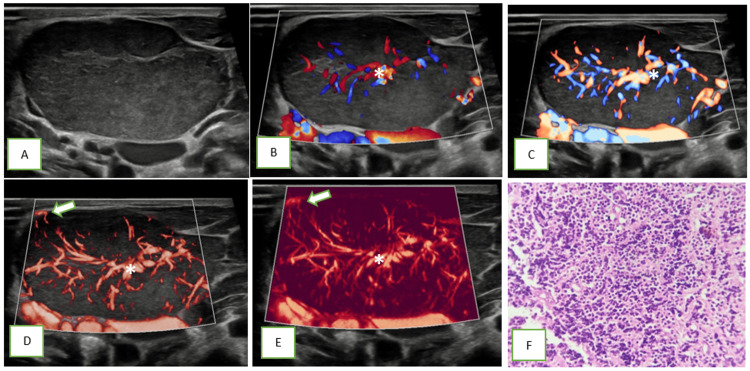
Non-Hodgkin lymphoma. A 60-year-old patient presented with multiple enlarged bilateral cervical lymph nodes. On cross-sectional imaging, the patient also had multiple enlarged mediastinal and abdominal lymph nodes. (A) Gray-scale US showed an enlarged lymph node with disturbed normal nodal architecture. (B-E) Color Doppler, power Doppler, MVI (color), and MVI (power), respectively, demonstrated central (*) vascularity in increasing order (from B to E), with peripheral neovascularization detected only by MVI (white arrow in D and E, highest by power mode (E)), suggesting neoplastic etiology. (F) Corresponding cytology showed atypical lymphoid cells with hyperchromatic nuclei and increased cellularity, consistent with non-Hodgkin lymphoma. US: ultrasound; MVI: microvascular imaging

An enlarged cervical lymph node in the case of non-Hodgkin lymphoma in Figure 3 showed disturbed normal nodal architecture with Doppler evaluation showing mixed central (*) and peripheral vascularity with progressively improved visualization of vascular flow from conventional Doppler to MVI. Fine irregular neovascularization was appreciated only on MVI (white arrow in Figures 3D, 3E), highlighting its superior sensitivity for detecting abnormal tumor-related microvascularity suggestive of neoplastic etiology.

**Figure 4:**
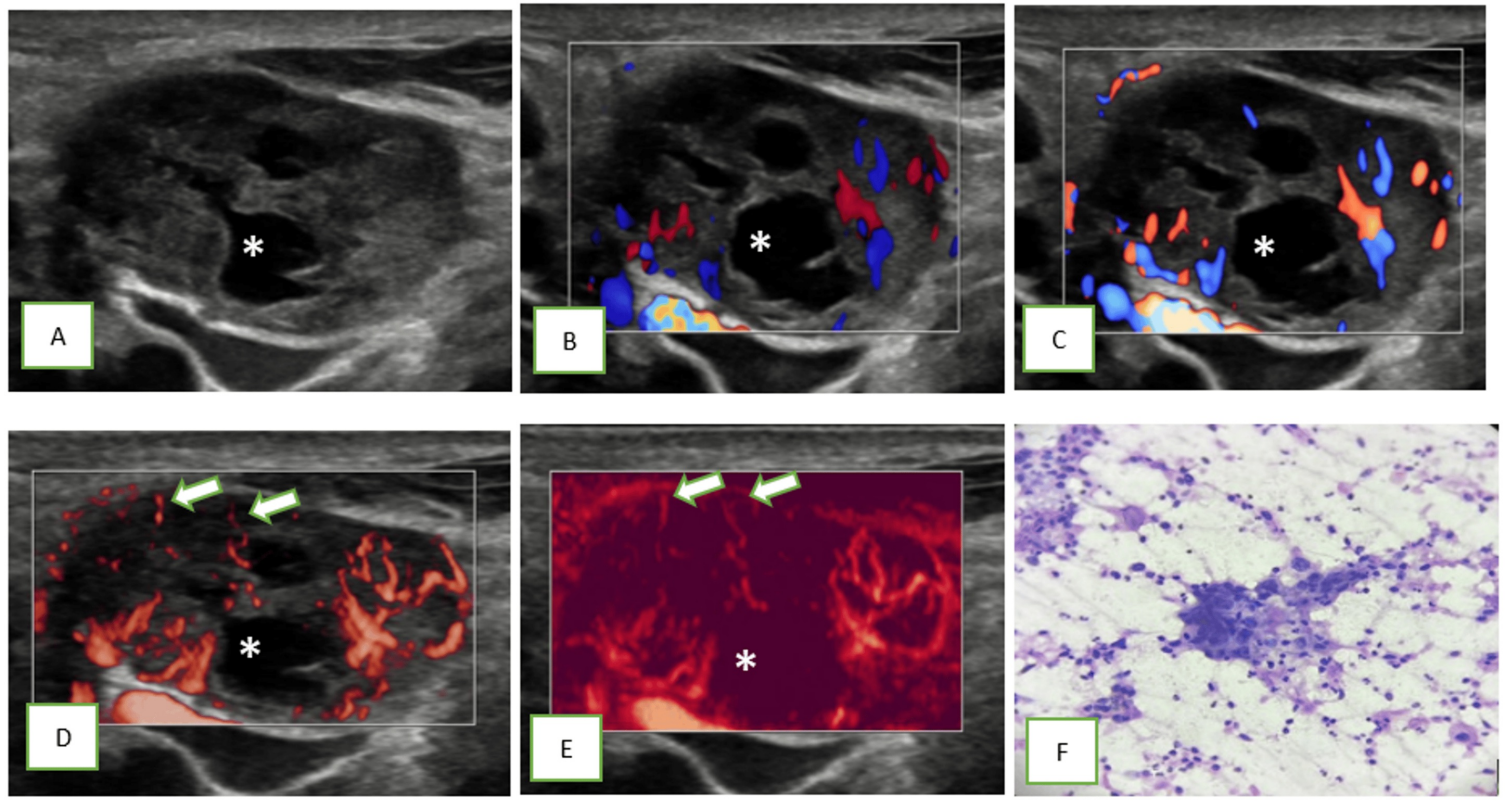
Metastatic deposits of poorly differentiated squamous cell malignancy. A 55-year-old man with a history of biopsy-proven squamous cell carcinoma of the base of the tongue presented with palpable cervical lymph nodes for 7-8 months. (A) Gray-scale ultrasound (US) image showing an enlarged heterogeneous cervical lymph node with central necrotic areas (*) and loss of normal hilar architecture. (B, C) Color Doppler and power Doppler, respectively, show distorted peripheral vascularity with a necrotic area (*). (E, F) MVI (color) and MVI (power) modes, respectively, demonstrated enhanced irregular peripheral neovascularization (white arrows) with distorted microvascular architecture with necrotic areas (*)-likely suggesting metastatic malignant etiology. (F) FNAC shows clusters of atypical pleomorphic malignant squamous cells with hyperchromatic nuclei and keratinous debris, consistent with metastatic deposits of poorly differentiated squamous cell malignancy. MVI: microvascular imaging; FNAC: fine-needle aspiration cytology

**Figure 5:**
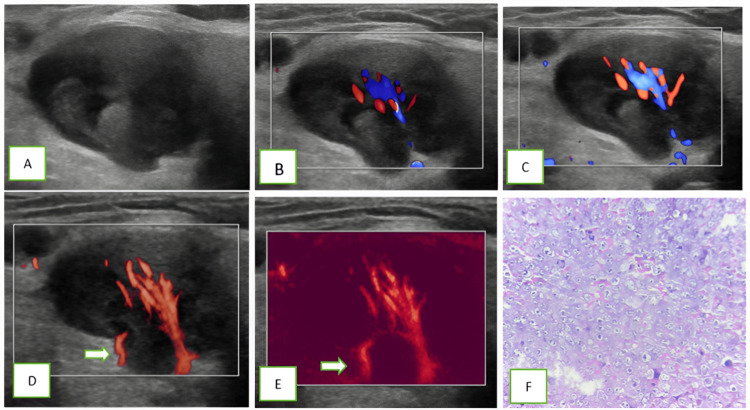
Metastatic deposits from poorly differentiated epithelial malignancy. An elderly chronic smoker presented with breathlessness, abdominal pain, and cervical lymphadenopathy for 6-8 months. (A) Gray-scale ultrasound (US) image showing an enlarged cervical lymph node with loss of normal hilar architecture and cortex. (B, C) Color Doppler and power Doppler, respectively, revealed hilar vascularity. (E, F) MVI (color) and MVI (power) modes, respectively, demonstrated peripheral neovascularization (white arrows)-likely suggesting metastatic malignant lymphadenopathy. (F) FNAC shows clusters of atypical malignant epithelial cells with pleomorphic hyperchromatic nuclei, consistent with metastatic deposits from poorly differentiated epithelial malignancy. MVI: microvascular imaging; FNAC: fine-needle aspiration cytology

Malignant metastatic cervical lymph nodes on ultrasound typically appear as heterogeneous nodes with loss of normal hilar architecture and areas of necrosis. In a case of metastatic deposits from poorly differentiated squamous cell malignancy (Figure 4), Doppler evaluation (Figures 4B-4E) demonstrated predominantly peripheral vascularity with only MVI enabling visualization of irregular neovascularization (white arrows in Figures 4D, 4E) that was not conspicuous on color and power Doppler techniques. Similarly, Figure 5 shows a case of metastatic deposits from poorly differentiated epithelial cell malignancy, where neovascularization (white arrows in Figures 5D, 5E) was identified exclusively by MVI, highlighting its added value and improved diagnostic confidence in characterizing malignant metastatic lymph nodes.

## Discussion

Ultrasound is the first-line imaging modality for evaluating cervical lymph node diseases because of its availability, real-time assessment, and lack of ionizing radiation. However, gray-scale ultrasound features such as nodal size, shape, and echotexture often overlap between inflammatory and neoplastic conditions, limiting diagnostic accuracy. Therefore, assessment of vascular flow patterns using Doppler-based techniques plays a crucial complementary role in lymph node characterization [1].

Benign lymph nodes usually show central or hilar vascularity, whereas malignant nodes tend to demonstrate peripheral or mixed vascular patterns due to tumor-related neoangiogenesis and capsular invasion [2]. Color Doppler ultrasound (CDUS) enables assessment of intranodal vascular distribution. Despite its utility, CDUS has limited sensitivity for detecting slow-flow and microvascular signals, which may lead to underestimation of malignant vascularity [3]. PDI improves sensitivity to low-velocity blood flow and reduces angle dependency, allowing better visualization of overall vascular density. However, its susceptibility to motion artifacts and lack of directional flow information may reduce diagnostic specificity [4]. MVI provides a superior depiction of peripheral and irregular microvascular flow patterns in metastatic cervical lymph nodes compared with conventional Doppler techniques and thus improves diagnostic accuracy in identifying malignant nodal involvement due to enhanced visualization of tumor-related neovascularity and low-velocity vascular flow signals [5].

By suppressing motion artifacts while preserving true blood flow signals, MVI allows detailed visualization of low-velocity microvasculature without contrast agents [6,7]. It improves the detection of tumor neovascularity, inflammatory hyperemia, and organ perfusion.

Ryoo et al. reported that microvascular ultrasonography better differentiated metastatic from tuberculous lymphadenopathy by improving visualization of vascular architecture and internal vascularity [8]. Similarly, Lee et al. found that MVI improved the characterization of indeterminate cervical lymph nodes and achieved high diagnostic accuracy for detecting metastatic involvement [9].

MVI is also useful in characterizing focal lesions in the liver, thyroid, breast, and kidney [10]. Only a limited number of studies in the literature have evaluated the diagnostic performance of MVI in comparison with conventional Doppler techniques, particularly in morphologically indeterminate cervical lymph nodes, and have reported the superiority of MVI.

A comparative summary of selected studies from the literature evaluating the diagnostic performance of MVI in cervical lymphadenopathy is presented below, highlighting their principal findings and observed advantages over conventional Doppler techniques (Table 4).

**Table 4:** Comparative analysis of previous studies assessing microvascular imaging (MVI) in cervical lymphadenopathy. US: ultrasound

SR. NO.	Study name	Study focus	Key findings	Clinical implication
1.	Present study (2026)	Evaluation of cervical lymph nodes using gray-scale US, color Doppler, power Doppler, and MVI	Reactive nodes demonstrated preserved hilar vascularity with orderly branching, whereas malignant nodes showed increased peripheral and mixed vascularity, better visualized on MVI	Improved detection of peripheral neovascularity and malignant vascular patterns compared with conventional Doppler techniques
2.	Li et al. (2020) [1]	Systematic review and meta-analysis of superb MVI (SMI) for lymph node evaluation	SMI demonstrated high diagnostic accuracy in differentiating benign from malignant lymph nodes by detecting low-velocity microvascular flow	Supports the role of SMI as a reliable non-invasive imaging modality for nodal characterization
3.	Sim et al. (2019) [2]	SMI for differentiation of benign and malignant cervical lymph nodes	Malignant nodes showed significantly increased peripheral and mixed vascularity patterns compared with benign nodes	SMI improves the characterization of nodal vascular architecture and assists in the differentiation of malignant lymphadenopathy
4.	Yang et al. (2024) [3]	Comparison of Doppler imaging and MVI in cervical lymph node blood flow analysis	MVI provided superior visualization of slow-flow intranodal vessels with fewer blooming and motion artifacts	Improves confidence and sensitivity in the assessment of nodal vascularity
5.	Huang et al. (2024) [5]	SMI for metastatic cervical lymph node evaluation in papillary thyroid carcinoma	Metastatic nodes showed increased peripheral and chaotic microvascular flow patterns better visualized on SMI	Improves diagnostic accuracy for identifying metastatic nodal involvement
6.	Ryoo et al. (2016) [8]	Utility of microvascular ultrasonography in differentiating metastatic lymphadenopathy from tuberculous lymphadenitis	Metastatic lymph nodes demonstrated increased internal vascularity and displaced central vessels, whereas tuberculous lymph nodes more frequently exhibited avascular patterns. Microvascular US detected these vascular differences more effectively than conventional power Doppler	Enhanced visualization of intranodal microvascular architecture improves differentiation between metastatic and inflammatory lymphadenopathy
7.	Lee et al. (2020) [9]	Role of MVI in triaging indeterminate cervical lymph nodes in papillary thyroid carcinoma	Metastatic lymph nodes showed peripheral and aberrant vascular signals on MVI, which were strongly associated with malignant involvement. MVI demonstrated high specificity and overall diagnostic accuracy	Improved identification of metastatic cervical lymph nodes and better characterization of indeterminate nodal lesions compared with conventional US assessment

Our study showed that MVI provided superior depiction of lymph nodal vascularity, enhancing visualization in both inflammatory and neoplastic etiologies, with clearer demonstration of peripheral and aberrant neovascularization in neoplastic lymph nodes. Possible reasons for few false-negative MVI findings could be early or micrometastatic nodal involvement without significant neoangiogenesis, markedly low-flow tumor vessels below the detection threshold, extensive necrosis reducing detectable vascularity, and deep-seated nodes with vascular signals affected by attenuation or suboptimal insonation. Also, operator-dependent technical factors may contribute to missed malignant lesions.

Identification of a greater number of malignant lymph nodes on MVI ultrasound improves lesion characterization by identifying abnormal microvascular patterns (neovascularization). This enhanced visualization gives direction to further management and helps in selecting the most suspicious nodes for targeted FNAC or biopsy, prioritizing nodes for further imaging, and guiding closer surveillance, thereby aiding subsequent clinical decision-making.

Study limitation

The study was limited by its single-center design, relatively small sample size, small study duration, small malignant subgroup, and lack of statistical assessment of interobserver agreement. Future multicentric studies with larger cohorts are needed to validate and standardize MVI protocols and explore quantitative vascularity assessment methods.

## Conclusions

Color Doppler, power Doppler, and MVI are effective ultrasound modalities for the evaluation of cervical lymphadenopathy. Among these, MVI provides superior visualization of slow-flow and peripheral microvascular patterns, enabling improved non-invasive differentiation between inflammatory and malignant cervical lymph nodes. When integrated with conventional Doppler techniques, MVI enhances visualization of intranodal vascular architecture, increases diagnostic confidence, and assists in identifying the most suspicious nodes for targeted FNAC or biopsy, prioritizing nodes for further cross-sectional imaging, and guiding focused follow-up. This has the potential to reduce multiple or repeated invasive procedures by guiding high-yield lymph nodes for FNAC and serves as a valuable adjunct in routine sonographic assessment and clinical decision-making in patients with cervical lymph node disease.
